# MRI-Guided Cardiac Catheterization in Congenital Heart Disease: How to Get Started

**DOI:** 10.1007/s11886-022-01659-8

**Published:** 2022-02-02

**Authors:** Elena K. Amin, Adrienne Campbell-Washburn, Kanishka Ratnayaka

**Affiliations:** 1 Division of Pediatric Cardiology, UCSF Benioff Children’s Hospitals, University of California, San Francisco, San Francisco, CA USA; 2 Division of Intramural Research, National Heart, Lung, and Blood Institute, National Institutes of Health, Bethesda, MD USA; 3 Division of Pediatric Cardiology, Rady Children’s Hospital, University of California, San Diego, 3020 Children’s Way, San Diego, CA USA

**Keywords:** Interventional cardiac magnetic resonance, Magnetic resonance imaging, Cardiac catheterization, Congenital heart disease

## Abstract

**Purpose of Review:**

Cardiac magnetic resonance imaging provides radiation-free, 3-dimensional soft tissue visualization with adjunct hemodynamic data, making it a promising candidate for image-guided transcatheter interventions. This review focuses on the benefits and background of real-time magnetic resonance imaging (MRI)-guided cardiac catheterization, guidance on starting a clinical program, and recent research developments.

**Recent Findings:**

Interventional cardiac magnetic resonance (iCMR) has an established track record with the first entirely MRI-guided cardiac catheterization for congenital heart disease reported nearly 20 years ago. Since then, many centers have embarked upon clinical iCMR programs primarily performing diagnostic MRI-guided cardiac catheterization. There have also been limited reports of successful real-time MRI-guided transcatheter interventions. Growing experience in performing cardiac catheterization in the magnetic resonance environment has facilitated practical workflows appropriate for efficiency-focused cardiac catheterization laboratories. Most exciting developments in imaging technology, MRI-compatible equipment and MRI-guided novel transcatheter interventions have been limited to preclinical research. Many of these research developments are ready for clinical translation.

**Summary:**

With increasing iCMR clinical experience and translation of preclinical research innovations, the time to make the leap to radiation-free procedures is now.

## Introduction

Since its inception, cardiac catheterization has been performed with X-ray guidance to guide movement of wires, catheters, and deployment of devices in the heart [1]. This imaging guidance is suboptimal. It provides visualization of radio-opaque objects (wires and devices) within the patient’s X-ray which only provides gross detail of the cardiac silhouette, trachea, bronchi and bony structures rather than blood vessels or cardiac anatomy. Vessels and intracardiac structures must be delineated by direct injection of iodinated contrast to perform angiography of the blood pool and the surrounding soft tissue is inferred. The inherent nature of this imaging modality exposes the patient and operators to radiation associated health risks and operator orthopedic injuries associated with wearing protective lead [2–4]. Adjunctive non-radiating ultrasound and echocardiographic guidance have proven useful and convenient. Nevertheless, it is inadequate for full procedural guidance of complex interventions. Radiation-free, real-time cardiac MRI (CMR) with 3-dimensional (3D) soft tissue imaging and superior hemodynamic data has potential to be the preferred catheterization laboratory imaging modality of the future.

## Why Pursue iCMR for Congenital Heart Disease?

CMR catheterization was first introduced nearly 20 years ago [5]. The logic behind migration toward CMR guidance is compelling. First, CMR provides excellent soft tissue visualization, 3-dimensional (3D) reconstructions of complex anatomy, and delineation of surrounding structures. CMR also permits characterization of tissue properties, such as edema, fibrosis, and iron content. For the congenital heart disease population, the superior soft tissue imaging afforded by CMR catheterization is of great importance given the frequency with which complex congenital and post-surgical anatomy is encountered. This is not only beneficial for anatomic catheter navigation. It may optimize diagnostic and interventional procedures such as improved endomyocardial biopsy yield using an MRI-compatible and visible bioptome and better ablation lesion visualization during real-time MRI-guided electrophysiology studies [6–9, 10•]. Second, CMR hemodynamics, namely cardiac function and blood flow, are better than traditional X-ray catheterization-derived hemodynamics. CMR-derived hemodynamics, particularly those involving complex shunt calculations, are more accurate than traditional Fick-derived calculations for congenital heart disease patients [11, 12]. CMR also offers useful hemodynamics not available with traditional X-ray such as regurgitant valve fractions and volumetric or flow analysis of any chamber or vessel. Real-time CMR-guided diagnostic right, and in some cases left, heart catheterization has been performed with similar procedure times to X-ray–guided procedures with the added benefit of additional and superior hemodynamic data from cardiac MRI [11, 13•, 14, 15••, 16]. Physiologic provocation states including vasodilator testing with inhaled nitric oxide, intravenous fluid or inotrope challenge and exercise study with MRI-compatible bicycle are feasible as part of real-time CMR-guided cardiac catheterization, while maintaining accuracy of hemodynamic evaluation [11, 14, 15••]. Third, CMR catheterization is radiation-free in growing and developing children with congenital heart disease that face true radiation exposure risk [2]. Fourth, interventional CMR offers the opportunity of real-time visual assessment of transcatheter interventions and deployed devices on the surrounding valves, vessels and extracardiac structures in addition to the hemodynamic effect of the intervention [17, 18].

### Pre-clinical

Early experience was focused on preclinical proof of concept work and novel interventions taking advantage of CMR. Interventions commonly performed by pediatric and adult congenital interventional cardiologists under X-ray and echocardiographic guidance have been reported in animal models for many years including pericardiocentesis, balloon angioplasty, balloon valvuloplasty, atrial septal defect device closure, pulmonary artery stent angioplasty, coarctation stent angioplasty, transcatheter aortic valve replacement [17–23]. Real-time CMR guidance may allow more precise diagnostic studies as shown in improved targeted biopsy yield with real-time CMR guidance compared with X-ray [6]. Real-time CMR-guided chemoablation targeting the conductive isthmus between scarred ventricular myocardium regions susceptible to ventricular tachycardia is promising [24]. Moreover, real-time CMR-guidance may facilitate novel transcatheter interventions such as those that require transthoracic access to the heart or transcatheter vessel anastomosis: closed-chest percutaneous transthoracic ventricular septal defect (VSD) device closure, closed-chest percutaneous transthoracic ventricular access, posterior percutaneous transthoracic left atrium access for mitral valve interventions and transcatheter cavopulmonary shunt [25–29]. These preclinical demonstrations have illustrated the significant promise of CMR-guidance.

### Clinical

In recent years, multiple new and long standing interested groups have pushed forward building and growing clinical diagnostic CMR catheterization programs [5, 12, 13•, 14, 15••, 16]. Indications include pulmonary hypertension, post-heart transplant surveillance, cardiomyopathy, intracardiac shunt, congenital heart disease, and Fontan fenestration test occlusion [12, 15••, 30–32]. With cumulative experience, workflow solutions to the challenges of the MRI environment have been developed. After an initial learning curve, CMR-guided catheterization times even with adjunct MR imaging and MR hemodynamic data are comparable to traditional X-ray guided cardiac catheterization [5, 11, 13•, 14, 15••]. Video examples of real-time CMR-guided catheterization for congenital heart disease patients, with refined workflow and efficient process, are available at the online links detailed in Table 1. Clinical real-time CMR-guided electrophysiology studies and successful radiofrequency ablation experience continues to grow [33, 34].

**Table 1:** Online resources

**Link**	**Content**
https://www.youtube.com/watch?v=7JxBNIoJLrQ	- Real-time MRI-guided right and transeptal left heart catheterization at Children’s National Heart Institute, Washington, DC
https://icmr.nhlbi.nih.gov	- Presentations from the iCMR workshops 2015 – 2019 at the Society for Cardiovascular Magnetic Resonance Scientific Sessions
https://www.optoacoustics.com/medical/imroc-ir	- Opto-acoustics IMROC IR Wireless communication with demonstration of real-time MRI-guided right heart catheterization (conference proceedings, Society for Cardiovascular Magnetic Resonance Scientific Sessions 2017)
https://nhlbi-mr.github.io/PRiME	- PRIME (Physiologic Recording in MRI Environment) system
https://www.ncbi.nlm.nih.gov/pmc/articles/PMC5585983/bin/12968_2017_374_MOESM3_ESM.tif	- “Postage stamp” views to pre-select prior to real-time imaging guidance (supplement to [29] PMID: 28874164 [15••])

Clinical interventional CMR has encountred slow progress. In humans, there have been limited reports of successful transcatheter CMR-guided interventions including pulmonary balloon valvuloplasty and aortic coarctation angioplasty [20, 35]. The limiting factor to more widespread clinical translation of real-time MRI-guided interventions has primarily been availability of MRI-compatible transcatheter devices approved for clinical use.

## How to Start an iCMR Program

### Team Building

Most important to start and successfully run an iCMR program is recruiting an invested, experienced, enthusiastic and adaptable team. Key team members are detailed in Table 2. The cardiac catheterization team will require introduction to working in the MR environment and the MRI team will require acquaintance to cardiac catheterization to gain mutual understanding of the combined iCMR environment. CMR technologists require training and practice to refine real-time protocols and switching between pre-selected views required for real-time guidance of interventional procedures [36, 37]. Reaching out to other services who may wish to use an interventional MRI-suite including interventional radiology, neurosurgery and other surgical sub-specialties can help to build momentum and expand applications to benefit overall patient care [38]. The entire team should be invited to give input on design of potential iCMR facilities. Institutional programatic investment is crucial.

**Table 2:** Suggeted iCMR team members

Cardiac catheterization team
*- Interventional cardiologist with experience in congenital heart catheterization* *- Assistants (nurse practitioners, fellows, or additional physicians)* *- Catheterization laboratory nurses* *- Catheterization laboratory technologists*
Cardiac MRI team
*- Imaging specialist: CMR imaging trained pediatric cardiologist or radiologist* *- MRI technologists: At least one technologist to train in real-time image guidance protocols* *- MRI physicists* *- MRI nurse*
Procedural clinical and administrative support team
*- Pediatric cardiac anesthesia team with experience of patient care in the MRI-environment* *- MRI safety officer* *- Vendor representative* *- Cardiac catheterization and MRI administrative staff (for procedure scheduling)* *- Other services with potential interest in performing MRI-guided procedures (pediatric cardiac electrophysiology, interventional radiology, neurosurgery, and other surgical subspecialties)*

*CMR*, cardiac magnetic resonance; *MRI*, magnetic resonance imaging

### Starting with Limited Facilities and Resources

**Fig. 1:**
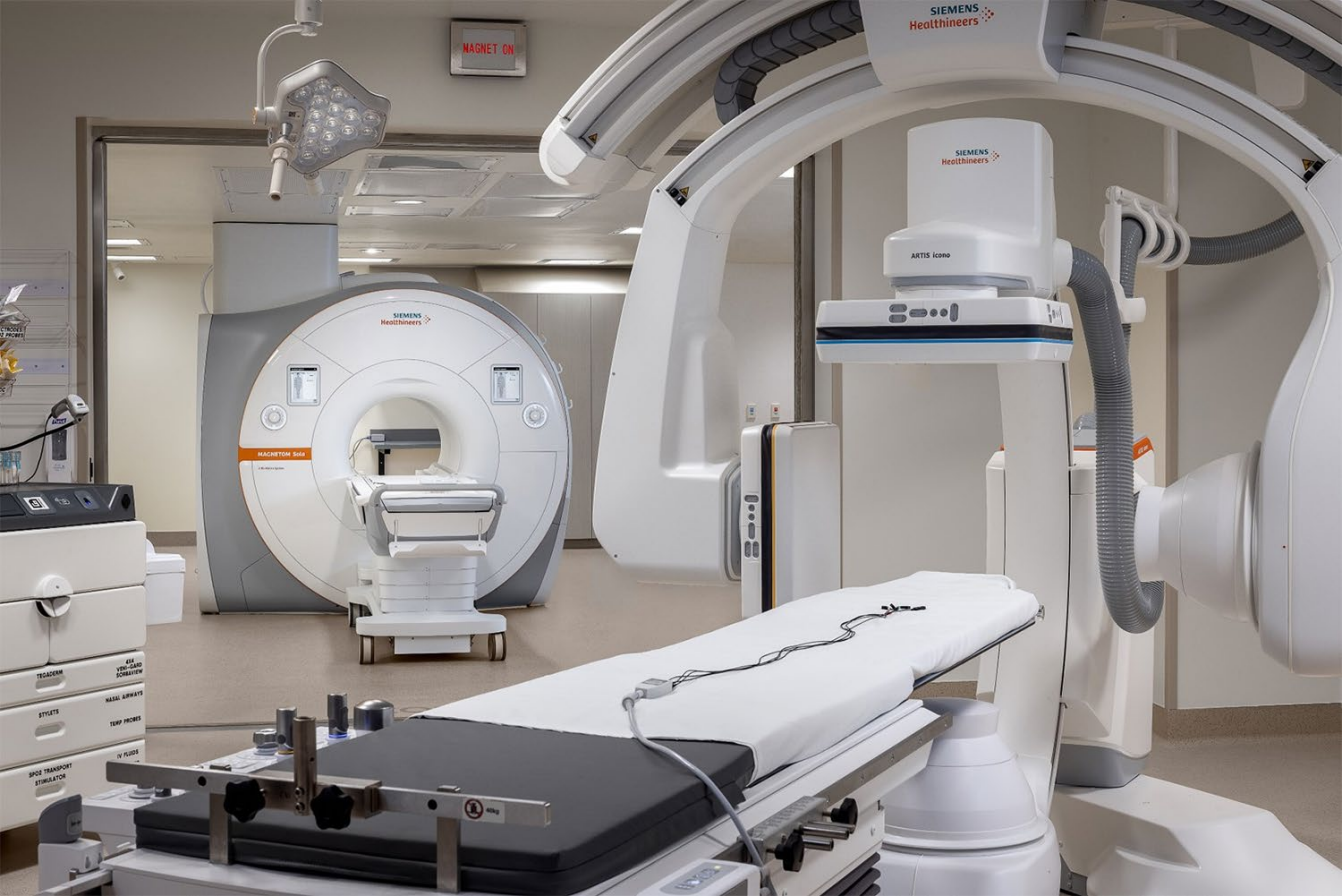
iCMR suite at Rady Children’s Hospital, University of California - San Diego. A co-localized biplane X-Ray fluoroscopy room and 1.5T cardiac MRI room are separated by radiofrequency shielded sliding doors allowing independent and combined room use. A second biplane X-Ray fluoroscopy room is adjoining a room to house a future imaging modality also intended for independent and combined room use

Some centers have proactively planned and built iCMR suites (typically with a biplane X-Ray room adjoining a MRI room separated by shielded sliding doors for independent or combined operation; Fig. 1). Other centers have taken advantage of upcoming hospital construction to plan iCMR suites. The costs associated with building a new iCMR suite, particularly when hospital construction is not already planned, may be prohibitive. Many centers must garner enthusiasm, consensus and experience by initiating experience with existing limited facilities. For example, centers with only a 3T magnet may not be able to achieve ideal real-time cardiac imaging precluding guidance of catheters within the heart and equipment is more susceptible to heating at higher magnet power. Centers with only 3T scanners can still reduce radiation exposure and invasive procedure time by performing most diagnostic evaluation via CMR including flow and shunt calculations with superior anatomic delineation of cardiac structures compared to angiography, followed by invasive X-ray cardiac catheterization to obtain direct pressure measurements and perform transcatheter interventions. Similarly, invasive catheters can be pre-placed under X-ray guidance before transfer to MRI. Initially performed at 1.5T, this has been referred to as “MRI-augmented” catheterization [39]. This type of combined sequential CMR and invasive cardiac catheterization evaluation initiates the process of working together as an iCMR team increasing familiarity of all involved parties with both modalities. The likely increase in CMR volume may also help to advocate for institutional funding of further iCMR related resources. Another starting point for iCMR programs is embracing 3D CMR-derived overlay. Ideally obtained immediately before same setting X-ray cardiac catheterization, CMR overlays directly onto live X-ray fluoroscopy serve as a roadmap with the potential to simplify complex transcatheter interventions, decrease radiation exposure/contrast burden/procedure time, add diagnostic value, increase operator confidence, and enable novel procedures [40–43].

### Where to Perform iCMR Procedures: Purpose-built Suites versus Adapting Existing MRI Rooms

The major consideration for centers embarking on an iCMR program is whether to use existing diagnostic CMR facilities to start performing real-time CMR-guided procedures or to purpose build an iCMR suite with adjacent combined X-ray fluoroscopy and CMR rooms (Fig. 1) [44•, 45]. Centers in the process of hospital construction and designing new facilities can use that opportunity to advocate for X-ray and CMR rooms to be co-localized. To perform iCMR procedures, the CMR room is ideally located adjacent or in proximity to a room suitable for patient preparation and may require non-MRI-compatible equipment prior to patient transfer into the CMR room. Once in the CMR room, there must be adequate space for patient monitoring and hemodynamic recording equipment, projection screens or monitors and sterile table setup with cardiac catherization equipment next to the MRI scanner. As with conventional X-ray–guided cardiac catheterization, the operator must be able to view live real-time CMR during device navigation in addition to the contemporaneous hemodynamic data while performing the procedure. This can be achieved by installation of shielded projector systems or LCD monitors in the CMR room (Table 3) and demonstrated in the online resource link to recorded CMR-guided cases (Table 1) [10•, 15••]. The risk of unstable arrhythmia during congenital cardiac catheterization is rare but a response plan must be in place. MRI-conditional defibrillators are under development, but not yet approved for clinical use [46]. The preparation room or another CMR adjacent room, such as the X-Ray fluoroscopy portion of an iCMR suite, must be available for patient evacuation from the CMR room for pacing or defibrillation in case of unstable arrhythmia or code [44•]. Proximity of the CMR-room to the X-ray fluoroscopy room limits time taken to transfer the patient between modalities for any X-ray dependent interventions. Patient transfer, often with multiple infusion lines and ventilator tubing, is simplified by iCMR suites with adjoining CMR and X-ray fluoroscopy rooms in a straight line configuration. Radiofrequency-shielded sliding doors (Table 2, Fig. 1), between the X-ray fluoroscopy and CMR rooms in a combined iCMR suite maintain the ability for efficient patient transfer between rooms. Importantly, it allows for simultaneous use of the MRI room for diagnostic studies when non iCMR cardiac catheterization cases are scheduled in the X-ray fluoroscopy room. This optimizes clinical use of the suite. Costs can preclude ability to build a dedicated iCMR only suite in centers with limited space and funding. Mobile MRI systems eliminating the need for patient transport exist primarily for neuroimaging applications [47]. Theoretically attractive for iCMR procedures, programs exploring dual use to date have found roadblocks in practicality for iCMR procedures and additive costs unattractive. A growing number of centers have reported successful diagnostic focused iCMR programs using existing CMR rooms. After installing required iCMR hardware and software (Table 3), this is a viable option to get an iCMR program off the ground [13•].

**Table 3:** Infrastructure and equipment to start an iCMR program. Commercially available products are listed to share knowledge of available resources used by centers referenced in this review. This does not reflect product endorsement, the authors do not have conflicts of interest with any companies, and other examples may be available

Equipment/infrastructure	Examples
1.5 T wide-short-bore scanner optimized for cardiovascular imaging	- Vendor specific
CMR surface coils for cardiovascular imaging	- Vendor specific
Vendor specific real-time scanner control and visualization software	- RTHawk by GE - Monte Carlo prototype software by Siemens, - iSuite by Philips
In-room display	
- Shielded projector systems - LCD monitor	- Gaven Industries - Sentient
In-suite cameras	- Gaven Industries
Audio communication noise cancelling headsets	- IMROC by OptoAcoustics
Patient monitoring	- Expression MRI Patient Monitoring System, Invivo Corporation
Hemodynamic recording system augmentation	- Physiological Recording in MRI Environment [PriME] system - Wireless telemetry system, PELEX-MAX, PinMed Inc
Nitric oxide delivery system	- INOmax, Mallinckrodt Pharmaceuticals
Electrophysiology equipment specific to MRI-guided arrhythmia ablation	
- Recording system and simulator	- Advantage MR EP Recorder/Simulator, Imricor Medical Systems, USA
- Ablation catheter	- Vision MR^TM^ ablation catheter, Imricor Medical Systems
Sterile procedure maintenance in the MRI-room	- Large sterile drapes for MRI-scanner - Long extension tubing for infusions - MRI-compatible stand to serve as a procedure table
Commercially available “off the shelf” standard catheterization equipment that is also MR-compatible	
- Balloon wedge catheter and 1% dilute gadolinium solution for balloon inflation	- Arrow balloon wedge catheter, Teleflex Medical
Purpose built MRI-conditional catheterization equipment	
- MR-conditional guidewire	- Angled-tip Emeryglide MRWire, Nano4Imaging

### Imaging Hardware and Software

Wide, short-bore MRI scanners facilitate operator proximity to reach vascular access sites for cardiac catheterization. This is particularly relevant in the setting of increased ergonomic challenges with children and infants who may be completely within the central bore of the scanner [15••, 38]. The major imaging vendors each have their own MRI scanner options suitable for iCMR applications that can accommodate rapid real-time imaging that demands fast gradient hardware and dedicated phased-array torso coils, including coils for pediatric patients. In addition, each of the major imaging vendors provides accompanying software (Table 3) to facilitate multi-planar or 3D volume imaging, and interactive sequence modification to aid real-time MRI-guided procedures. MRI scanners are not specifically designed for the procedural environment but can be easily draped in a straightforward manner to create sterile conditions suitable for cardiac catheterization [15••, 36]. The current recommended magnet strength for clinical iCMR procedures is 1.5T providing excellent temporal and spatial resolution despite expected cardiorespiratory motion at fast heart rates in the pediatric population [10•]. Image acquisition by cardiac MRI designed for diagnostic data collection over multiple heartbeats does not translate well for real-time imaging guidance. iCMR procedures require rapid image acquisition, reconstruction and display to visualize catheters moving through the heart in an acceptable timeframe for procedural guidance. Real-time imaging typically uses balanced steady state free-precession (bSSFP) for suitable blood-myocardium contrast, magnetization preparation pulses for improved contrast, and parallel imaging reconstruction for imaging speed. The capability to interactively modify slice orientation and position, as well as other imaging parameters, during continuous imaging is critical for procedural guidance with real-time MRI. With multiple imaging protocol and reconstruction modifications, real-time magnetic-resonance imaging has progressed to facilitate guidance of up to ten frames per second which is comparable to modern conventional X-ray guidance but with superior soft tissue data with single or multiplanar viewing options [10•, 15••, 37]. Visibility of MRI-compatible equipment in the heart and vessels with real-time imaging is often limited without modifications to the equipment and imaging parameters as detailed in the discussion of cardiac catheterization equipment below. Dilute gadolinium contrast for balloon inflation of commercially available balloon wedge catheters together with imaging pre-pulses make this contrast filled balloon more visible in real-time imaging in comparison to the blood pool [10•, 48]. This is one example of a simple equipment and imaging parameter modification to aid in facilitating iCMR clinical translation.

### Patient Monitoring and Hemodynamics

MRI-conditional anesthesia equipment, ventilators, infusion pumps/tubing and patient monitoring equipment perform well for routine diagnostic MRI clinical practice. This equipment can be used for iCMR procedures with pre-planned consideration for ideal placement on the patient and location in the room. During iCMR procedures, limited patient access in the MRI bore, dimmed room lights, and sterile coverage drapes necessitates special consideration for optimal access to intravenous lines and patient airway [49]. iCMR studies require clear electrocardiogram and invasive blood pressure tracings for patient monitoring and interpretation of pressure tracings. MRI-compatible hemodynamic recording systems can be outfitted with signal optimization to filter noise and signal distortion caused by MRI scanning and allow for clinically acceptable electrocardiogram and invasive blood pressure tracings (Table 3) [50]. MRI-conditional nitric oxide delivery systems are available as detailed in Table 3. There are also unique patient considerations. Personal patient pumps, relevant to pulmonary hypertension patients receiving subcutaneous treprostinil do not have long tubing and are not MRI compatible. Infusions must be discontinued for the duration of time in the MRI room if clinically acceptable or switched to the intravenous route.

### Communication

While operators can enjoy being free from the weight and constraints of wearing lead, they have to contend with the loud noise emitted by the MRI scanner necessitating noise cancelling protective headsets. These headsets can serve a dual purpose, when paired with an MR-compatible microphone, allowing communication between the operators, anesthesia team, MR-technologist and team in the control room. Wireless solutions are optimal. Potential communication systems and examples of use are available in online video links (Table 1). There must also be a separate communication channel between the patient and the MRI control room and cardiac catheterization operators for patients undergoing procedures under conscious sedation.

### Patient Safety Considerations

Widespread implementation of routine iCMR has been challenging for many reasons including the inherent hurdles of working in the magnetic resonance environment and limited availability of MRI-compatible equipment for cardiac catheterization. MRI scanners generate a magnetic field powerful enough to turn standard medical equipment into dangerous projectiles such as oxygen tanks. Equipment specifically designed for routine anesthesia care in the magnetic field is commonplace including MRI-compatible nitric oxide delivery systems. Cardiac catheterization equipment primarily composed of metal are susceptible to becoming projectiles when pulled toward the magnet. Typical standard cardiac catheterization wires, catheters and device delivery systems are susceptible to significant heating under real-time CMR-guidance. Safety protocols and standard operating procedures can easily mitigate these risks. One example is if anesthesia induction, vascular access or any portion of the catheterization case is performed prior to entering the CMR room, a time-out metal safety check should be performed prior to patient transfer to the CMR room. This guarantees that all non-compatible objects, such as stainless-steel wires and needles, have been removed from the field. Likewise, and similar to non-iCMR CMR cases, each team member entering the CMR room should perform a personal check to make sure they are not inadvertently carrying or wearing items likely to become projectiles. The radio-frequency field risk of the MRI environment can also generate currents that interfere with pacemakers and with hemodynamic monitoring systems. As per routine for any diagnostic MRI scan, the compatibility of the patient’s existing implanted devices is determined prior to approving the study on an individual case basis. Consideration should be given to suturing in place any vascular access sheaths prior to transfer to the CMR room as transfer between the X-ray fluoroscopy and CMR rooms introduces the potential risk of sheath dislodgement [15••]. Frequent simulations and drills for routine and emergency clinical scenarios including cardiac arrest in the iCMR environment can reduce the risk of patient-safety related events [44•, 49].

### Cardiac Catheterization Equipment

Much of the standard equipment for a cardiac catheterization is MRI-compatible including most sterile drapes, transducers and tubing, access sheaths and non-braided diagnostic catheters such as balloon wedge catheters. Availability of specifically designed MRI-compatible and MRI-visible equipment to facilitate real-time MRI-guided cases is continually increasing. A brief summary of useful existing equipment, including specific MRI-guided ablation procedure equipment, is listed in Table 3. There are limitiations in the MRI-visibility of most standard catheterization wires, catheters, sheaths and devices. In addition, long metallic devices such as guidewires and braided catheters are susceptible to radiofrequency-induced heating during real-time MRI and should be avoided [51, 52]. Cardiac catheteterization equipment used in CMR-guided procedures is classed as passive or active depending on method of visualization. Passive device visualization relies on intrinsic device properties such as air or Gadolinium filled balloons (balloon wedge catheters) or signal voids created by metallic devices, for example nitinol guidewires. Some purpose built devices for MRI-guided interventions have additional passive markers, such as iron oxide bands, for signal void creation and improved MRI visualization. An MR-conditional approved guidewire with passive markers exists and has been reported for iCMR procedures in patients with congenital heart disease [53]. A much larger number of approved MR-conditional devices is needed to expand the potential diagnostic and interventional reach of iCMR procedures. Active devices incorporate electronic components connected to the scanner such that devices themselves can receive local MRI signal for improved visualization, for example, generating a bright-colored overlay to highlight the device on real-time imaging [6, 24–29]. Clinical translation of these active devices has been limited. Radiofrequency receiver coils can be embedded into the device to allow for active tracking of the device during imaging rather than relying on manually adjusting to the exact slice plane that shows the device and have been used for MRI-guided arrhythmia studies [9].

### Future Directions

**Fig. 2:**
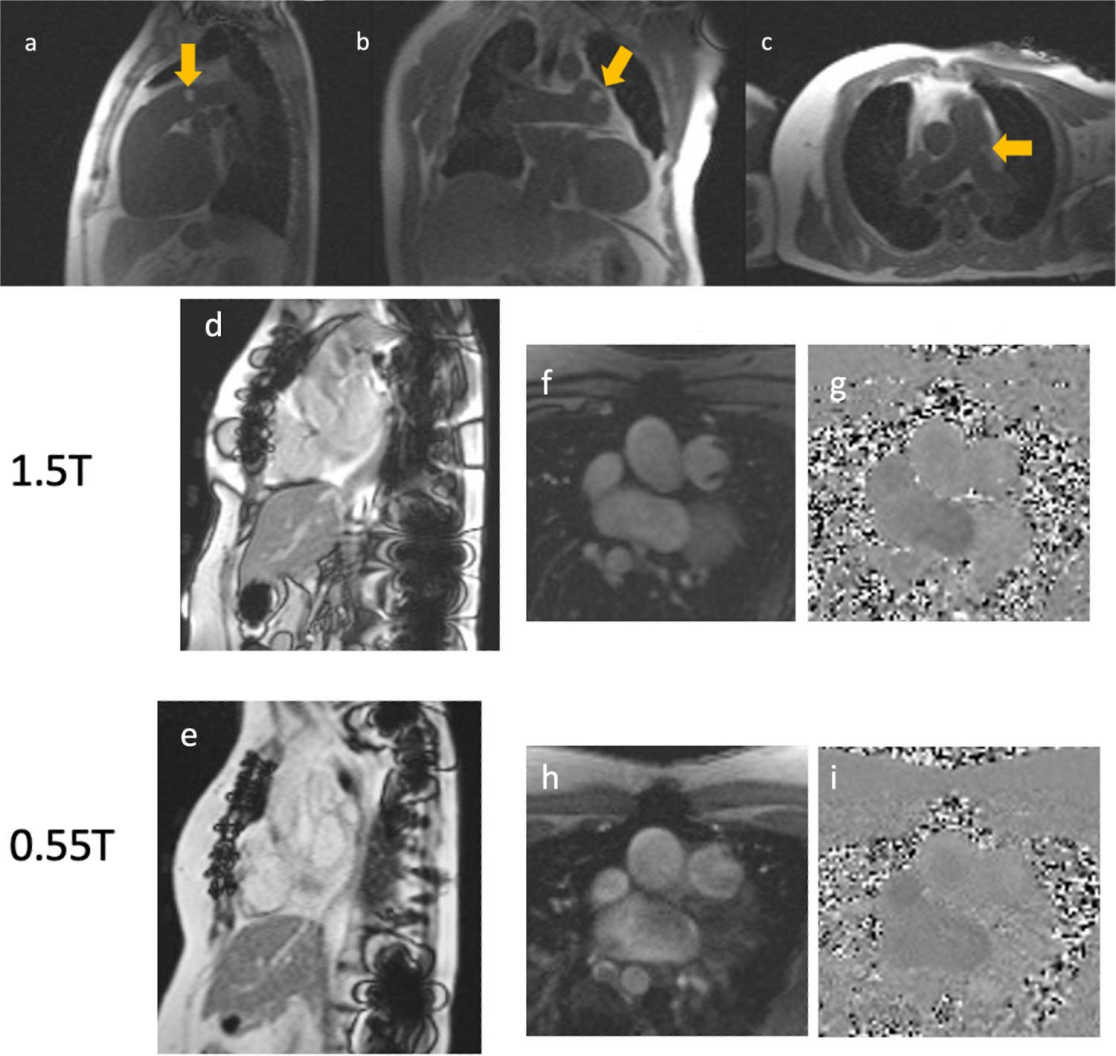
Low-field CMR catheterization. Low field, 0.55T, real-time MRI-guided right heart catheterization using commercially available metallic hydrophilic coated guidewire (invisible with partial saturation pulse) and gadolinium filled commercially available balloon (*yellow arrows*) tipped wedge catheter in the sagittal (**a**), coronal (**b**) and axial (**c**) planes. For patients post cardiac interventions with metallic devices and implants including sternal wires, as illustrated for a patient post tetralogy of Fallot repair (**d**–**g**), metal artifact at 1.5T (**d**) is reduced at 0.55T (**e**) without compromising the ability to determine flow via the turbulent pulmonary valve at 0.55T (**h** and **i**) compared to 1.5T (**f** and **g**)

Rather than rely on development of purpose-built equipment for the MRI environment, advances in imaging may be a better route to widespread iCMR feasibility. Modification of imaging techniques has made it possible to investigate off-label use of commercially available X-ray equipment such as hydrophilic glidewires at 1.5T for real-time MRI-guided right heart catheterization with visualization and without significant heating [54, 55]. Lower field MRI, for example the modification of a commercial 1.5T scanner to operate at 0.55T, as demonstrated in Fig. 2, is an exciting advance for iCMR procedures explored in the last few years [56]. At this lower magnet strength, even with preferred real-time imaging techniques, there is less heating of catheterization equipment traditionally used for X-ray-guided cardiac catheterization. Low field imaging may rapidly expand the equipment available for use in iCMR procedures from the armamentarium of existing traditional cardiac catheterization equipment, while also reducing artifacts caused by post-intervention metallic implants and maintaining diagnostic CMR image quality [56–58]. With these advances in imaging technology in addition to purpose-modified MR compatible devices, CMR-guidance could dramatically increase the scope of transcatheter congenital heart interventions particularly those that require visualization of soft tissues such as anastomosis of vessels and other procedures currently undertaken solely by open heart surgery.

## Conclusions

Cardiac magnetic resonance is an appealing modality for congenital heart disease cardiac catheterization image guidance as it provides radiation-free 3D soft tissue visualization and additional hemodynamic data for complex congenital anatomy and interventions. Multiple review articles have been published over the last two decades on the promise and progress of iCMR for standard and future transcatheter interventions to replace existing suboptimal X-ray imaging guidance with associated patient and occupational hazards [59–65]. Nevertheless, despite the promise and compelling logic supporting iCMR catheterization, clinical translation has been slow. There has been growing experience building iCMR suites and working in the magnetic resonance environment at multiple centers around the world. With continued experience of starting and running iCMR programs, building consensus and knowledge sharing in conjunction with translation of preclinical research innovations, iCMR could potentially facilitate routine radiation-free transcatheter interventions to become the standard operating modality of the future. The time to make the leap away from radiation emitting procedures is now.
